# A Modified Artificial Bee Colony Algorithm for *p*-Center Problems

**DOI:** 10.1155/2014/824196

**Published:** 2014-01-29

**Authors:** Alkın Yurtkuran, Erdal Emel

**Affiliations:** Department of Industrial Engineering, Uludag University, Görükle Campus, 16059 Bursa, Turkey

## Abstract

The objective of the *p*-center problem is to locate *p*-centers on a network such that the maximum of the distances from each node to its nearest center is minimized. The artificial bee colony algorithm is a swarm-based meta-heuristic algorithm that mimics the foraging behavior of honey bee colonies. This study proposes a modified ABC algorithm that benefits from a variety of search strategies to balance exploration and exploitation. Moreover, random key-based coding schemes are used to solve the *p*-center problem effectively. The proposed algorithm is compared to state-of-the-art techniques using different benchmark problems, and computational results reveal that the proposed approach is very efficient.

## 1. Introduction

The *p*-center problem, which is also known as the minimax location-allocation problem, is one of the best-known NP-hard problems in the field of logistics and discrete facility location [1]. This problem consists of locating *p* facilities (centers) on a network such that the maximum of the distances between nodes and their nearest centers is minimized. In the *p*-center problem, *n* nodes (customers) and distances between nodes are given, and centers should be located at any given node. The *p*-center problem can be used in a variety of real-life applications such as locating fire stations, police departments, or emergency centers.

The *p*-center problem is NP hard [2], and the number of feasible solutions is $\left(\begin{array}{c} N\\ C \end{array}\right)$, where *N* is the node number and *C* is the number of centers. Various heuristics have been proposed to solve the *p*-center problem [3–5]. Moreover, exact algorithms that can solve the problem in certain conditions have been proposed [4, 6, 7].

The *p*-center problem has attracted increasing attention in recent years. D. Chen and R. Chen [1] proposed new relaxation techniques for the solution of *p*-center problems. Mladenović et al. [8] presented a basic variable neighborhood search and two tabu search heuristics for the *p*-center problem without triangle inequality. Caruso et al. [9] proposed a metaheuristic algorithm, called Dominant, in which a series of set-covering problems according to a predefined maximum distance are solved. Pacheco and Casado [10] proposed a new scatter search-based approach for the *p*-center problem. Davidović et al. [11] introduced an improved bee colony algorithm (BCO), proposing a new concept based on improving the complete solution held by each bee, instead of applying constructive BCO; moreover, they showed that the proposed algorithm produces high-quality solutions within negligible CPU times.

The artificial bee colony (ABC) algorithm, which is a biologically inspired population-based metaheuristic algorithm, was recently introduced for continuous function optimization by Karaboga [12]. Due to its simplicity and ease of implementation, the ABC algorithm has been extensively applied to both continuous and discrete optimization problems since its invention. Various comparison studies, in which the ABC algorithm was compared to novel metaheuristic algorithms, such as particle swarm optimization (PSO), differential evolution (DE), and genetic algorithm (GA), have been performed to show its effectiveness [13, 14]. These studies show that the ABC algorithm outperforms other novel algorithms on several instance problems.

However, several studies have shown that the ABC algorithm is good at exploration but poor at exploitation. The local search equation, in which a new neighbor solution is generated, has received extensive attention because it controls the exploration-exploitation balance. Therefore, to improve the performance of the algorithm, various modifications to the search equation have been proposed in the literature. Zhu and Kwong [15] proposed a global best guided ABC algorithm, which additionally uses the global best information in the search equation as in PSO. Inspired by DE, Gao and Liu introduced a modified version of the ABC algorithm in which ABC/Best/1 and ABC/Rand/1 were employed as local search equations [16, 17]. Li et al. [18] presented a modified version of ABC (IABC) in which the best-so-far information, inertial weight, and acceleration coefficient are used when employing local search. Furthermore, Kang et al. [19] proposed the Rosenbrock ABC algorithm, which combines Rosenbrock's rotational method and the original ABC algorithm. Singh [20] used the ABC algorithm to solve the leaf-constrained minimum spanning tree problem. To improve exploration, Alatas [21] employed chaotic maps as initialization and chaotic searches as a local search equation. Gao et al. [22] proposed a new search equation and used Powell's method as a local search technique. Xu et al. [23] described a new ABC (NABC) algorithm that employs a modified version of the DE/Best/1 strategy. Akay and Karaboga [24] proposed a modified version of the ABC algorithm in which a control parameter that determines the number of parameters to be modified during the production of a neighboring solution is introduced. Moreover, an adaptive technique for reduction of step size was also used.

Although the ABC algorithm was originally developed for solving continuous optimization problems, the use of the ABC algorithm for combinatorial problems has attracted extensive attention in recent years. Kashan et al. [25] presented DisABC for solving binary optimization problems. In DisABC, a measure of dissimilarity between binary vectors is used instead of vector subtraction, which is used in the original ABC algorithm's search equation. Szeto et al. [26] proposed a modified ABC algorithm for capacitated vehicle routing problems and introduced various operators to produce new neighbor solutions. Uzer et al. [27] used a hybrid ABC-support vector machine mechanism for feature selection and classification for medical databases. Alvarado-Iniesta et al. [28] used the ABC algorithm for the optimization of the material flow in a manufacturing plant. Ji et al. [29] proposed a modified ABC algorithm for solving 0-1 multidimensional knapsack problems and introduced a novel communication mechanism based on the updating and diffusion of inductive pheromones produced by bees. Furthermore, numerous papers have used the ABC algorithm for scheduling problems, such as the lot-streaming flow shop scheduling problem [30], total flow time minimization in permutation flow shops [31], the flexible job-shop scheduling problem [32], and the no-idle permutation flow shop scheduling problem with total tardiness criterion [33]. Readers can refer to Karaboga et al. [34] for an extensive literature review of the ABC algorithm and its applications.

In this paper, a modified ABC algorithm (M-ABC) is proposed to solve well-known *p*-center problems. The M-ABC algorithm uses random key-based encoding for solution representation and employs a new multisearch strategy in which different search strategies are used for generating new neighbor solutions. The remainder of this paper is organized as follows: Section 2 gives the mathematical model for the *p*-center problem, Section 3 covers the traditional ABC algorithm, and the proposed algorithm is described in Section 4. Computational results are given in Section 5, and finally, Section 6 concludes the paper.

## 2. Mathematical Formulation

The *p*-center problem can be briefly defined as follows. Let *N* = {1,2, 3,…, *n*} be the node set, and there is a distance *d*_*ij*_ associated with each node pair (*i*, *j*) ∈ *N*,  *i* ≠ *j*. The objective is to locate *p* centers, = {1,2, 3,…, *p*}, at given nodes so that the maximum distance between a node and its nearest facility *p* is minimized. The *p*-center problem can be mathematically formulated as follows [11].

Decision variables are as follows:

(1) $$\begin{array}{c} x_{ij}=\{\begin{array}{cc} 1, & \text{if}\;\text{node}\;i\;\text{is assigned}\;\text{to}\;\text{center}\;\text{at}\;\text{node}\;j,\\ 0, & \text{otherwise}, \end{array}\\ c_{j}=\{\begin{array}{cc} 1, & \text{if}\;\text{node}\;\;j\;\text{is}\;\text{the}\;\text{center},\\ 0, & \text{otherwise}. \end{array} \end{array}$$

Parameters are as follows:

 
*N*: set of nodes,
 
*d*_*ij*_: travel time (distance) from node *i* to *j*, where *i*, *j* ∈ *N*,
 
*p*: number of centers.
  (2) Minimize   ∑i∈N ∑j∈Ndij∗xij
  (3) subject  to ∑j∈Nxij=1, ∀i∈N,  i≠j,
  (4) xij≤cj, ∀i,j∈N,  j≠i,
  (5) ∑j∈Ncj=p, ∀j∈N,
  (6) xij,yj∈{0,1} ∀i,j∈N.

The objective function represents the minimization of the largest distance between a node and its closest facility. Constraint (3) expresses that each node should be assigned to a center. Constraint (4) prevents any center from being located outside the node set. The total number of located facilities is set to *p* by constraint (5).

## 3. Artificial Bee Colony Algorithm

The ABC algorithm is a population-based metaheuristics algorithm that mimics the foraging behavior of honey bee swarms. The ABC algorithm classifies bees in a colony into three main groups: employed bees, onlooker bees, and scout bees. Employed bees are responsible for exploiting the food sources and sharing the information about these food sources. Onlooker bees wait in the hive and take the food source information from employed bees to make a decision on further exploiting the food source. Scout bees randomly search the environment to find a new food source.

In the ABC algorithm, each candidate solution to the problem is associated with a food source and is represented by an *n*-dimensional real-coded vector. The quality of a solution corresponds to the nectar amount on that food source, and one employed bee explores each food source. In other words, the number of the employed bees is equal to the number of food sources. The colony is equally divided into employed and onlooker bees. A food source, which cannot be improved for a predetermined number of tries, is abandoned and the employed bee associated with that food source becomes a scout. In the ABC algorithm, the employed and onlooker bees are responsible for exploiting, whereas the scout bees handle exploring.

The main steps of the ABC algorithm are as follows [12]:

1. initialization,
2. evaluating the population,
3. repeat,
4. employed bee phase,
5. onlooker bee phase,
6. scout bee phase,
7. until (termination criteria are satisfied).

### 3.1. Initialization

In the initialization step, the ABC algorithm generates a randomly distributed population of SN solutions (food sources), where SN denotes the number of employed or onlooker bees. Let *x*_*i*_ = {*x*_*i*,1_, *x*_*i*,2_,…, *x*_*i*,*D*_} represent the *i*th food source, where *D* is the problem size. Each food source is generated within the range of the boundaries of the parameters by

(7) $$\begin{array}{c} x_{i,j}=x_{j}^{\min}+\text{rand}\left(0,1\right)\left(x_{j}^{\max}-x_{j}^{\min}\right), \end{array}$$

where *i* = 1,…, SN, *j* = 1,…, *D*. *x*_*j*_^min⁡^, and *x*_*j*_^max⁡^ are the lower and upper bound for the dimension *j*, respectively.

### 3.2. Employed Bee Phase

In the employed bee phase, employed bees generate a neighboring food source *v*_*i*_ by performing a local search around each food source *i* ∈ {1,2,…, SN} as follows:

(8) $$\begin{array}{c} v_{i,j}=x_{i,j}+\Phi_{i,j}\left(x_{i,j}-x_{k,j}\right), \end{array}$$

where *j* is a random integer in the range [1, *D*] and *k* ∈ {1,2,…, SN} is randomly chosen food source that is not equal to *i*. Φ_*i*,*j*_ is a random number in the range [−1,1]. A greedy selection is applied between *x*_*i*_ and *v*_*i*_ in which the better solution will be retained. Then, employed bees will return to their hive and share the information on new solutions with onlooker bees.

### 3.3. Onlooker Bee Phase

Onlooker bees select a food source depending on the probability value prob associated with that food source. The value *p* is calculated as follows:

(9) $$\begin{array}{c} {\text{prob}}_{i}=\frac{f_{i}}{\sum_{j=1}^{\text{SN}}f_{j}}, \end{array}$$

where *f*_*i*_ is the objective function value of solution *i*. By using this mechanism, food sources having better fitness values will be more likely to be selected. Once the onlooker bee has chosen the food source, she generates a new solution using (7). As in the employed bee phase, a greedy selection is carried out between *x*_*i*_ and *v*_*i*_.

In the employed bee phase, a local search is applied to every food source, whereas only the selected food sources will be updated in the onlooker bee phase.

### 3.4. Scout Bee Phase

If a food source cannot be improved for a predetermined number of tries, then the employed bee associated with that food source becomes a scout bee. Then, the scout bee finds a new food source using (8). After the scout bee finds a new source, she becomes an employed bee again.

## 4. Proposed Artificial Bee Colony Algorithm

### 4.1. Random Key-Based Encoding

Because the ABC algorithm was first introduced for solving continuous optimization problems, real-coded solution vectors can be used directly in the calculation step of the objective function. To apply the ABC algorithm for combinatorial optimization problems like *p*-center problems, real-coded vectors should be converted to permutations of nodes. Therefore, in the proposed study, random key-based encoding is used to represent solutions.

The random key procedure was first introduced by Bean [35] for solving sequencing problems with genetic algorithms using real-coded genes. Snyder and Daskin [36] have also used random key-based encoding schemes to overcome the infeasibility problems in genetic algorithms. Moreover, numerous studies used random key-based encoding to convert real-coded vectors to feasible solutions [37–41].

In the proposed solution encoding, for any *n*-dimensional problem, when the real-coded solution values are sorted in a nondecreasing order, their corresponding indices (treated as centers) in the sorted order yield a solution. Original to the *p*-center problem, the number of centers is limited by the problem as *p*. Therefore the first *p* values of the sorted index vector are taken as the solution in this study. Once the center nodes are determined, the objective value is calculated as follows: max⁡_*i*∈*N*_⁡{min⁡_*j*∈*P*_⁡{*d*(*i*, *c*_*j*_)}} where *c* denotes the center nodes, *d* is the distance operator, *N* represents nodes set, and *P* = (1,2,…, *p*).

**Figure 1:**
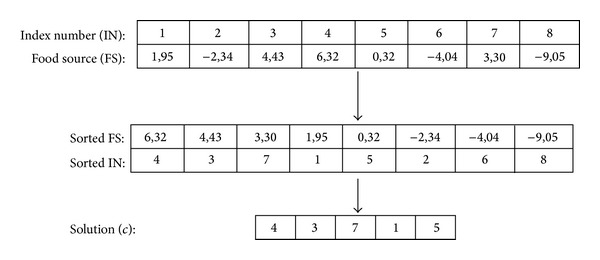
Example of random key-based encoding.

An example of this procedure is given in Figure 1 for a problem (*p* = 5, *n* = 8), where first the real-coded food source vector is sorted and then the first *p* indices of the sorted index vector are chosen as the centers.

### 4.2. Multisearch Strategy

The ABC algorithm has been shown to be good at exploration but poor at exploitation [15, 16]. Therefore, numerous studies have been performed to improve the performance of the ABC algorithm, and most of these studies investigated modifications for the search equation because it is the main part of the algorithm that controls the exploration-exploitation balance [15].

**Pseudocode 1:**
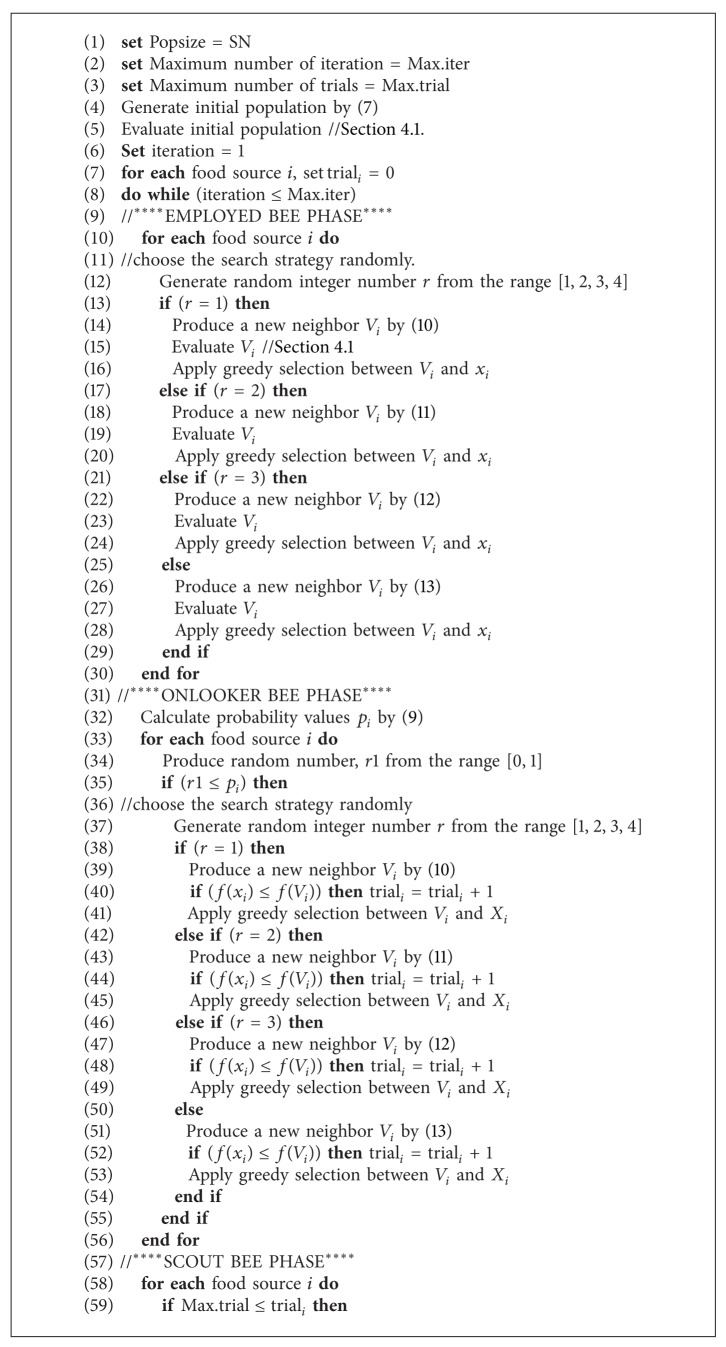

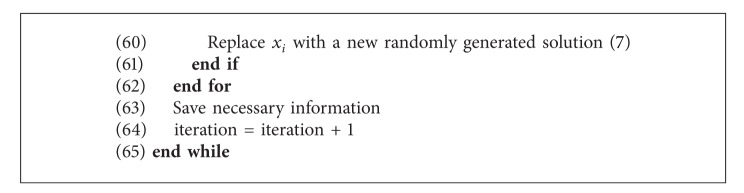
Pseudocode of M-ABC.

Each search strategy has distinctive characteristics and therefore may have an advantage or disadvantage in different stages of the overall search progress on different problems. In the present study, different search strategies taken from the literature are employed in a random manner to overcome this problem. In this paper, a new neighbor solution is generated using a search equation selected randomly from a candidate pool, which consists of selected search strategies. Four search equations are used, which are explained as follows.

(i) Original ABC [12]: described in detail in Section 3.2. The search equation is as follows:
  (10) $$\begin{array}{c} v_{i,j}=x_{i,j}+\Phi_{i,j}\left(x_{i,j}-x_{k,j}\right). \end{array}$$
(ii) Global best guided ABC [15]: inspired by PSO, Zhu and Kwong [15] described a new search equation to improve the exploitation and to take advantage of the global best solution information as follows:
  (11) $$\begin{array}{c} v_{i,j}=x_{i,j}+\Phi_{i,j}\left(x_{i,j}-x_{k,j}\right)+\Psi_{i,j}\left(y_{j}-x_{i,j}\right), \end{array}$$
  where *y*_*j*_ is the *j*th element of global best solution, Ψ_*i*,*j*_ is a uniform random number in [0, *C*] where *C* is a nonnegative constant and is suggested to be 1.5, Φ_*i*,*j*_ is a random number in the range [−1,1], and *j* ∈ {1,2,…, *n*} is a randomly chosen index.
(iii) ABC/best/1 [16]: based on the differential evolution algorithm, Gao and Liu [16] proposed a modified search equation as follows:
  (12) $$\begin{array}{c} v_{i,j}=x_{\text{best},j}+\Phi_{i,j}\left(x_{r1,j}-x_{r2,j}\right), \end{array}$$
  where the indices *r*1 and *r*2 are mutually exclusive integers randomly chosen from {1,2,…, SN} and different from the base index *i*; *x*_best_ is the best solution in the current population and *j* ∈ {1,2,…, *n*} is a randomly chosen index; and Φ_*i*,*j*_ is a random number in the range [−1,1].
(iv) ABC with Powell's method [22]: in order to improve the search ability and convergence speed of the search equation proposed in [15], Gao et al. [22] described a new search equation that uses the information of a randomly selected solution and the best solution as follows:
  (13) $$\begin{array}{c} v_{i,j}=x_{k,j}+\text{rand}\left(0,1\right)\left(x_{\text{best},j}-x_{k,j}\right), \end{array}$$
  where rand(0,1) denotes a uniformly distributed random number, *k* is an integer uniformly chosen from the range [1, SN] and is different from *i*, and *x*_best_ is the best solution in the current population.

### 4.3. Proposed Algorithm

Based on the explanations in the previous subsection, the general scheme of the M-ABC is given in Pseudocode 1.

## 5. Computational Results

In this section, the proposed M-ABC algorithm, which is discussed in the previous section, is analyzed in detail using several benchmarking problems. The M-ABC algorithm was coded using the C++ language executed on a computer with 2.00 GB RAM and an Intel Core2 Duo 2.00 GHz CPU. In order to evaluate the validity and success of M-ABC, the described algorithm is applied to 40 well-known benchmark problems that were originally developed for *p*-median problems [42]. These benchmark problems can be downloaded from ORLibrary at the following website: http://people.brunel.ac.uk/~mastjjb/jeb/orlib/pmedinfo.html. The problem parameters range from 100 to 900 and from 5 to 200 for the number of customers and the number of centers, respectively.

The proposed M-ABC approach is compared with the novel metaheuristic algorithms such as the improved bee colony algorithm (BCOi) [11], multistart interchange (M-I), variable neighborhood search (VNS), tabu search 1 (TS-1) and tabu search 2 (TS-2), proposed in [8], and scatter search (SS), proposed in [10]. To fairly compare the algorithms, the stopping criterion is taken as the maximum allowed CPU time (second) as in [8, 10, 11] and is set to one-tenth of the number of customers (i.e., *n*/10) for each problem. The parameters of the proposed algorithm have a remarkable effect on the quality and effectiveness of the algorithm. Therefore, with the help of the initial experiments, the search range is taken as [−10,10] and the population size and trial are set to 100 and 50, respectively, for all benchmarks.

The computational results for the benchmark problems are summarized in Table 1. In Table 1, the first column shows the problem name. The second and third columns denote the number of customers and the number of centers, respectively. The next six columns show the results from M-I, VNS, TS-1 and TS-2 [9], SS [10], and BCOi [11], and the last column represents the results with the M-ABC algorithm. In Table 1, the best result achieved from 10 consecutive runs is reported for each test problems similar to [9–11] and bold values denote best-known solutions found with the M-ABC algorithm. The ratio of the number of problems where the best solution is achieved, to the total number of problems solved is given in the last row of Table 1 for each algorithm.

As seen from Table 1, the M-ABC algorithm finds the best-known solutions for 37 out of 40 test problems. Results in Table 1 show that M-ABC, VNS, and TS-2 perform similarly in terms of solution quality. Furthermore, better results are obtained with M-ABC for pmed18 and pmed39, when compared to TS-2. M-ABC outperforms VNS for pmed39. It can be concluded from Table 1 that M-ABC is better than M-I, TS-1, and BCOi on most of the test problems. BCOi's performance is superior to that of M-ABC only on pmed30, where BCOi approach is the best performer among all algorithms. For the small size problems (*p* ≤ 10) SS and M-ABC yield the same values in terms of the best value; however it would not be a fair comparison since SS was not applied to bigger problems where *p* > 10 in [11]. In general, the results indicate that the M-ABC algorithm is superior to traditional and new metaheuristics.

For a better evaluation of metaheuristic algorithms, not only the solution quality but also the computation times should be investigated. However, it is not very easy to make an objective comparison between metaheuristics because both the programming languages and the machine configurations are not generally comparable, and in most studies, the complexities of the algorithms are not reported. Nevertheless, an approximate comparison can be made based on the million floating point operations per second (MFLOP) values of the processors on which the algorithms were coded and run [43].

The heuristics proposed in [8] was implemented on a Sun SPARCstation 10, and the SS algorithm in [10] was executed on an Intel Pentium III processor operating at 300 MHz. Moreover, both heuristics were coded in Pascal with the Delphi 5.0 compiler. BCOi in [11] was implemented on an Intel Core 2 Duo E6750 processor and was coded in the C programming language with the g++ compiler. The MFLOP values of the processor speeds based on the benchmark values obtained from the site http://www.netlib.org/benchmark/linpackjava/timings_list.html were used to normalize the CPU times. The MFLOP values and corresponding normalized CPU times spent on the benchmark problems for the algorithms are summarized in Table 2.

Table 2 shows the normalized CPU times with respect to the fastest CPU (Intel Core 2 Duo E6750) as the baseline. In Table 2, the CPU time in seconds spent by the M-ABC algorithm to achieve its best solution is reported like in [8, 10, 11]. Since M-ABC does not utilize any constructive initialization mechanism, it has a slower performance on the majority of small size test problems (*p* ≤ 10). On the other hand, M-ABC outperforms M-I, VNS, TS-1, and TS-2 on most of the test problems having *p* > 10 in terms of CPU time. As can be seen in Table 2, M-ABC is slightly slower than BCOi, which is the quickest algorithm in test algorithms.

## 6. Conclusion and Future Work

In this paper, a modified artificial bee colony (M-ABC) algorithm is presented to solve *p*-center problems. The proposed approach has two main contributions: random key-based encoding for solution representation and a new multisearch strategy in which different search strategies are employed in one overall search process. The M-ABC algorithm is compared to state-of-the-art metaheuristic algorithms with benchmark problems and is found to be effective and better than other algorithms in terms of both solution quality and CPU time on most of the benchmarks. The proposed M-ABC algorithm achieves best-known solutions for 37 out of 40 of the benchmark problems with competitive CPU times when compared to other metaheuristic algorithms. Future studies may include using adaptive selection for search strategies instead of random selection and application of the proposed approach on other real-life problems, such as vehicle routing or scheduling problems.

## Figures and Tables

**Table 1:** Computational results for benchmark problems.

Problem	*n*	*p*	M-I	VNS	TS-1	TS-2	SS	BCOi	M-ABC
Pmed1	100	5	127	127	127	127	127	127	**127**
Pmed2	100	10	98	98	98	98	98	98	**98**
Pmed3	100	10	93	93	93	93	93	93	**93**
Pmed4	100	20	74	74	74	74	—	74	**74**
Pmed5	100	33	48	48	48	48	—	48	**48**
Pmed6	200	5	84	84	84	84	84	84	**84**
Pmed7	200	10	64	64	64	64	64	64	**64**
Pmed8	200	20	58	55	55	55	—	55	**55**
Pmed9	200	40	46	37	37	37	—	37	**37**
Pmed10	200	67	30	20	20	20	—	20	**20**
Pmed11	300	5	59	59	59	59	59	59	**59**
Pmed12	300	10	51	51	51	51	51	51	**51**
Pmed13	300	30	41	36	36	36	—	37	**36**
Pmed14	300	60	36	26	26	26	—	27	**26**
Pmed15	300	100	29	18	25	18	—	18	**18**
Pmed16	400	5	47	47	47	47	47	47	**47**
Pmed17	400	10	40	39	39	39	39	39	**39**
Pmed18	400	28	29	28	28	35.71	—	29	30
Pmed19	400	80	28	19	23	19	—	19	**19**
Pmed20	400	133	26	14	22	14	—	14	**14**
Pmed21	500	5	40	40	40	40	40	40	**40**
Pmed22	500	10	40	38	38	38	38	39	**38**
Pmed23	500	50	30	23	23	23	—	23	**23**
Pmed24	500	100	25	16	18	16	—	16	**16**
Pmed25	500	167	22	12	24	12	—	12	**12**
Pmed26	600	5	38	38	38	38	38	38	**38**
Pmed27	600	10	33	32	32	32	32	32	**32**
Pmed28	600	60	25	19	19	19	—	19	**19**
Pmed29	600	120	23	13	23	13	—	14	**13**
Pmed30	600	200	20	11	19	11	—	10	11
Pmed31	700	5	30	30	30	30	30	30	**30**
Pmed32	700	10	30	29	29	29	29	29	**29**
Pmed33	700	70	22	16	16	16	—	16	**16**
Pmed34	700	140	21	12	20	12	—	12	**12**
Pmed35	800	5	30	30	30	30	30	30	**30**
Pmed36	800	10	28	27	27	27	27	28	28
Pmed37	800	80	23	16	22	16	—	16	**16**
Pmed38	900	5	29	29	29	29	29	29	**29**
Pmed39	900	10	24	24	23	24	23	24	**23**
Pmed40	900	90	21	14	22	14	—	14	**14**

Best/total			15/40	38/40	31/40	37/40	19/19	33/40	37/40

**Table 2:** CPU time comparison.

Problem	Normalized computational time (sec)						
	M-I (90 MFlop)	VNS (90 MFlop)	TS-1 (90 MFlop)	TS-2 (90 MFlop)	SS (50 MFlop)	BCOi (750 MFlop)	M-ABC (500 MFlop)
Pmed1	0,00	0,00	0,00	0,00	0,01	0,00	0,00
Pmed2	1,20	0,53	0,05	0,00	0,08	0,00	0,02
Pmed3	3,14	0,02	1,38	1,26	0,03	0,00	0,09
Pmed4	14,37	0,04	0,72	0,41		0,00	0,56
Pmed5	1,31	0,01	0,02	0,00		0,00	0,04
Pmed6	0,05	0,18	0,08	0,00	0,00	0,00	0,11
Pmed7	0,85	0,16	0,12	0,01	1,09	0,00	0,19
Pmed8	10,70	0,27	0,13	0,22		0,01	0,21
Pmed9	2,99	1,31	0,46	0,26		0,00	1,24
Pmed10	10,48	1,08	2,16	1,47		0,03	1,45
Pmed11	0,53	0,18	0,24	0,47	0,53	0,00	0,33
Pmed12	68,39	0,65	0,17	1,36	0,29	0,02	0,56
Pmed13	6,26	1,23	17,49	0,16		0,01	1,20
Pmed14	19,89	8,48	12,56	4,66		0,14	2,09
Pmed15	66,01	6,70	0,93	0,76		0,04	3,69
Pmed16	0,25	0,01	0,01	0,00	0,16	0,00	0,51
Pmed17	17,13	3,12	0,18	0,03	0,70	0,01	0,96
Pmed18	6,56	14,35	2,32	0,00		0,15	1,63
Pmed19	53,73	36,01	2,63	14,53		0,07	2,04
Pmed20	75,78	26,23	17,86	6,27		0,09	4,33
Pmed21	0,35	0,15	0,01	0,00	0,19	0,00	0,86
Pmed22	13,41	9,92	5,57	4,29	5,41	0,19	1,44
Pmed23	10,50	13,50	1,77	1,00		0,23	2,01
Pmed24	1,45	31,74	29,58	3,30		0,08	2,59
Pmed25	3,65	21,08	0,04	27,21		0,38	2,44
Pmed26	0,07	0,07	0,27	0,01	0,30	0,00	1,02
Pmed27	9,42	0,61	0,04	0,10	5,24	0,01	1,47
Pmed28	27,67	3,01	91,77	10,56		0,05	2,73
Pmed29	1,12	91,49	0,01	18,15		1,14	2,04
Pmed30	100,29	23,63	0,01	10,16		0,35	4,98
Pmed31	4,15	0,07	0,03	0,02	0,15	0,00	0,81
Pmed32	14,09	19,83	3,25	10,15	1,43	0,06	2,12
Pmed33	59,70	96,81	58,98	11,53		0,72	2,06
Pmed34	6,33	19,22	0,01	9,70		0,39	3,68
Pmed35	1,87	0,80	0,22	1,58	0,33	0,01	1,34
Pmed36	9,89	12,72	7,07	6,07	1,76	0,08	2,98
Pmed37	9,38	143,74	0,04	11,88		0,12	2,77
Pmed38	0,42	0,23	0,05	0,10	0,75	0,00	0,18
Pmed39	155,73	0,72	168,12	0,66	2,37	0,01	1,29
Pmed40	1,26	59,25	0,01	5,73		0,19	2,06
